# Oxidative stress, biochemical, and histopathological changes associated with acute lumpy skin disease in cattle

**DOI:** 10.14202/vetworld.2022.1916-1923

**Published:** 2022-08-15

**Authors:** Ahmed Kamr, Hany Hassan, Ramiro Toribio, Anis Anis, Mohamed Nayel, Ali Arbaga

**Affiliations:** 1 Department of Animal Medicine and Infectious Diseases (Animal Internal Medicine), Faculty of Veterinary Medicine, University of Sadat City, Sadat City 32897, Egypt; 2 Department of Veterinary Clinical Sciences, College of Veterinary Medicine, The Ohio State University, Columbus, OH 43210, USA; 3 Department of Pathology, Faculty of Veterinary Medicine, University of Sadat City 32897, Egypt; 4 Department of Animal Medicine and Infectious Diseases (Animal Infectious Diseases), Faculty of Veterinary Medicine, University of Sadat City, Sadat City 32897, Egypt

**Keywords:** acute phase proteins, cytokines, lumpy skin disease, malondialdehyde, total anti-oxidant capacity

## Abstract

**Background and Aim:**

Lumpy skin disease (LSD) is a highly infectious endemic viral disease of cattle in Africa and the Middle East. The objectives of this study were to assess histopathological changes in cattle infected with LSD and measure serum malondialdehyde (MDA – oxidant) and total anti-oxidant capacity (TAC – anti-oxidant), trace elements (zinc, copper, and iron), cytokines (interleukin [IL]-1β, IL-6, and tumor necrosis factor-alpha [TNF-α]), haptoglobin (Hp), serum amyloid A (SAA), aspartate aminotransferase (AST), alanine aminotransferase (ALT), creatine phosphokinase (CPK), blood urea nitrogen (BUN), and creatinine concentrations.

**Materials and Methods:**

Blood samples were collected from a total of sixty native and mixed breed cattle; (healthy; n = 25) and (LSD diseased; n = 35). Serum concentrations of MDA and TAC were measured by colorimetric methods. Serum IL-1β, IL-6, IL-10, TNF-α, Hp, and SAA concentrations were determined using human-specific enzyme-linked immunoassay kits.

**Results:**

Serum MDA, cytokine (IL-1β, IL-6, and TNF-α), Hp, SAA, AST, ALT, CPK, BUN, and creatinine concentrations were significantly higher, while TAC, IL-10, zinc, copper, and iron concentrations were significantly lower in LSD compared to healthy cattle (p < 0.05). Cows and exotic mixed breed cattle were at higher risk of LSD oxidative stress than bulls and local breeds (p < 0.05). Age was not associated with the risk of LSD (p > 0.05). Histologically, there was extensive tissue necrosis, severe vasculitis, mononuclear cell infiltration, and intracytoplasmic inclusion bodies.

**Conclusion:**

LSD is associated with pro-oxidative and pro-inflammatory states from imbalances that favor pro-oxidant and pro-inflammatory factors in the detriment of anti-oxidant and anti-inflammatory factors, leading to organ dysfunction and ultimately death. Oxidative stress is more frequent in cows and mixed breed cattle than in bulls and local breeds.

## Introduction

Lumpy skin disease (LSD) is a highly infectious endemic viral disease of cattle in Africa, the Middle East, and Turkey. It is an emerging disease that in recent years has spread to some countries in Asia, the Balkans, and the Russian Federation [1]. It also affects water buffaloes and some wild ruminants, which could have epidemiological and economic implications related to disease dissemination and control. It is caused by the LSD virus (LSDV), also known as Neethling virus, a double-stranded DNA virus of the *Poxviridae* family that spreads by blood-sucking insects. This condition is characterized by fever, lethargy, and the sudden appearance of skin nodules that cover the entire body, mucous membranes, and internal organs [2–4]. Lesions in internal organs can lead to respiratory and gastrointestinal problems, including dyspnea, pneumonia, and ruminal atony [5]. Lumpy skin disease has economic implications due to weight loss, reduced milk production, abortions, infertility, damaged hides, and death [2–4].

Oxidative injury from excessive free radical production and reduced anti-oxidant capacity is central to the pathogenesis of multitude of acute and chronic disorders in different species [6]. Oxidative status is characterized by the elevation of oxidative biomarkers such as malondialdehyde (MDA) and a reduction of the anti-oxidant status, including total anti-oxidant capacity (TAC) in ruminants [7, 8]. Trace elements, including zinc, copper, and iron, are essential co-factors for anti-oxidant enzymes that protect cells against reactive oxygen species and free radical injury [7]. It has been shown that illness and inflammatory cytokines are associated with low copper and zinc concentrations in ruminants and deficiency of these elements could exacerbate oxidative injury in animals with evidence of systemic inflammation [7]. However, information on the oxidative status and trace elements with anti-oxidant properties in cattle with LSD remains to be investigated.

Increased concentrations of pro-inflammatory cytokines (e.g., interleukin [IL]-1β, IL-6, and tumor necrosis factor-alpha [TNF-α]) have been measured in cows with LSD during viremic states [9, 10]. IL-10 is an anti-inflammatory cytokine with immunomodulatory properties which could potentially be involved in the pathogenesis of LSD but the information on affected animals is lacking. The response to systemic inflammation in cattle is characterized by increases in haptoglobin (Hp) and serum amyloid A (SAA) (positive acute phase proteins) and decreases in albumin concentrations (negative phase protein), all produced by the liver [11, 12]. However, information on acute phase proteins and pro-inflammatory/anti-inflammatory cytokines in cattle with LSD is scarce. Cattle with LSD have altered liver, muscle, and renal function [9]. However, further information on biochemical markers will enhance our understanding of pathophysiology of bovine LSD. Histological features typical of LSD include histiocytic inflammation, necrotizing fibrinoid vasculitis of dermal blood vessels, and intracytoplasmic inclusion bodies in keratinocytes [13, 14]. In chronic lesions, there is necrosis of the dermis and epidermis, often leading to sloughing after 1–2 weeks. Superficial lymphadenopathy is also a consistent finding [13, 14].

The objectives of this study were to investigate oxidant, anti-oxidant, and inflammatory biomarkers as well as selected biochemical variables in cattle with LSD. We hypothesized that cattle with LSD will be in a systemic pro-oxidative and pro-inflammatory state from increased pro-oxidant factors and pro-inflammatory cytokines with reduction of anti-oxidants and anti-inflammatory cytokines.

## Materials and Methods

### Ethical approval

This study was approved by the Animal Ethics Committee at the Faculty of Veterinary Medicine, University of Sadat City (Approval code VUSC-007-1-21).

### Study period and location

This study was carried out from April 2021 to June 2021 at Menofia Governorate. The laboratory analysis was carried out at the Department of Animal Medicine and Infectious Diseases, University of Sadat City.

### Inclusion criteria

A total of sixty native and mixed breed cattle aged 2–3 years and of a different sex from the Menofia Governorate were divided into healthy (n = 25) and LSD affected (n = 35). Animals were fed silage and hay. The affected cattle were from a local outbreak of LSD. The healthy group included cattle from the distant farm (25 km) free of LSD. The diagnosis of LSD was made based on physical examination (fever, multiple skin nodules, and enlargement of multiple superficial lymph nodes), and confirmed by blood biochemistry and histopathological findings.

### Clinical examination

Historical information was gathered from all animals and the farm. A complete physical examination was performed on all animals, including attitude, body condition score (1−5), rectal temperature, heart rate, respiratory rate, and ruminal motility [15].

### Blood sampling

Five milliliters of blood were collected under aseptic jugular venipuncture from healthy and affected cattle and placed into plain serum clot tubes, allowed to clot for 1 h and centrifuged at 2000× *g* for 10 min. Serum was aliquoted into smaller volumes and stored at −80°C until biochemical analysis. A number of skin nodules were aseptically collected from each affected animal and stored at −20°C until DNA extraction.

### Molecular detection of LSDV

Detection of LSDV was performed by extracting DNA from skin nodules with a DNA extraction kit (QIAamp 51304, QIAGEN, Hilden, Germany) followed by polymerase chain reaction (PCR). Primers specific to the gene encoding the viral attachment protein of Capripoxviruses (5’-TTTCCTGATTTTTCTTACTAT-3’; 5’-AAATTATATACGTAAATAAC-3’) were used [16]. A 192 bp PCR fragment was consistent with the presence of the virus. A 25 μL PCR mixture containing 2.5 μL of 10 × PCR buffer, 1.5 μL of MgSO4 (25 mM), 2.5 μL of dNTPs (2 mM), 0.75 μL of the forward primer, 0.75 μL of the reverse primer, 1 μL of DNA template, 0.25 μL (0.25 IU) of KOD -Plus- Neo DNA polymerase (Toyobo Co., Osaka, Japan), and 15.75 μL of nuclease-free water. Cycling conditions were an initial cycle at 98°C for 2 min, and then 35 cycles of 98°C for 10s, 50°C for 30s, and 68°C for 10s and a final extension step of 68°C for 5 min.

### Oxidant, anti-oxidant, cytokine, copper, zinc, iron, acute phase protein, total protein (TP), albumin, aspartate aminotransferase (AST), alanine aminotransferase (ALT), creatine phosphokinase (CPK), urea, and creatinine concentrations

Serum concentrations of MDA and TAC were measured by colorimetric methods (Bio-Diagnostics Ltd, Egypt) [17, 18]. Serum IL-1β, IL-6, IL-10, TNF-α, Hp, and SAA concentrations were determined using human-specific enzyme-linked immunosorbent assay kits (Shanghai Coon Koon Biotech., Ltd; China). Intra and inter-assay coefficients of variations (CV) were calculated using bovine serum samples in duplicate (low and high concentrations). Intra- and inter-assay CV were < 15%, with good linearity (R^2^ = 0.92) [19]. Serum concentrations of trace elements (copper, zinc, and iron), liver and muscle enzymes (AST, ALT, and CPK), kidney function (blood urea nitrogen [BUN], and creatinine), TP, and albumin concentrations were measured by spectrophotometry using commercial kits (Bio-Diagnostics Ltd.) [20]. Serum globulin concentrations were assessed by subtraction of serum albumin from serum TP concentrations [21].

### Histopathological examination

Skin biopsies were collected from recently developed nodules and fixed in 10% neutral buffered formalin (pH 7.4) for 72 h, washed, dehydrated, embedded in paraffin wax, serially sectioned at 5 μm thickness and stained with hematoxylin and eosin for histopathological evaluation [22].

### Statistical analysis

Power analysis: Calculation of sample size per group was based on the number of animals required to achieve a power of 0.8, alpha = 0.05, using variables of interest MDA, TAC, acute phase protein, and cytokine concentrations. Data were assessed for normality by Shapiro–Wilk statistic, were normally distributed and expressed as means with standard deviations (SD) (mean ± SD). Comparison between the two groups was carried out by t-test. Associations between variables were determined by Pearson correlations (r). Oxidant/antioxidant, inflammatory/pro-inflammatory, and albumin/globulin (A/G) ratios were calculated for all animals. Serum variables were categorized into high, normal, and low concentrations based on cutoff values using 5–95% confidence interval (CI) from the healthy animals. Univariate analysis with odds ratios (OR) was determined for variables of interest. Receiver operating characteristic curve was used to determine sensitivity and specificity of measured parameters. Data were analyzed using SPSS version 16.0 (IBM Corporation, Armonk, NY, USA) and GraphPad Prism 8 (GraphPad, Inc., La Jolla, CA, USA). Significance was set at p < 0.05.

## Results

### Study population

Healthy cattle represented 41.7% (25/60) and cattle with LSD 58.3% (35/60) of the total population. Of the animals with LSD, 48.6% (17/35) were cows and 51.4% (18/35) were bulls. The mean age of healthy and affected cattle was 2.5 years old (2–3 years old). Age was not associated with susceptibility to LSD (p > 0.05). Of the affected cattle with LSD, 71.4% (25/35) were local breed and 28.6% (10/35) were exotic mixed breed.

### Clinical history and signs

Cattle with LSD had a history of lethargy, anorexia, and a low body condition score. Rectal temperature, heart, and respiratory rates were elevated while ruminal motility was decreased in LSD compared to healthy cattle (p < 0.05; Table-1).

**Table-1:** Clinical examination of healthy cattle and cattle with LSD (mean ± SD).

Variables	Healthy cattle (n = 25)	Cattle with LSD (n = 35)
Rectal temperature (°C)	38.7 ± 0.34	41.05 ± 0.69*
Heart rate (beats/min)	71.9 ± 2.02	86.6 ± 1.9*
Respiratory rate (breaths/min)	23.2 ± 1.5	40.2 ± 1.68*
Ruminal motility (movements/2 min)	4.1 ± 0.78	0.7 ± 0.48*
Body condition score	3.25 ± 0.28	1.5 ± 0.57*
Lethargy	No	Yes
Anorexia	No	Yes

LSD=Lumpy skin disease, SD=Standard deviation, n=Number,

* p < 0.05

### Molecular diagnosis of LSDV

**Figure-1:**
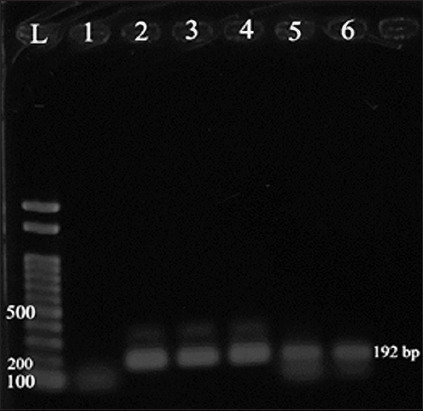
Electrophoretic pattern for lumpy skin disease virus-polymerase chain reaction assay, Lane L is 100 bp DNA ladder, Lanes 1 is negative. Lanes 2–6 are positive (amplicon size 192 bp).

The LSD infection was confirmed by PCR analysis of skin nodules extracted DNA using viral attachment protein-encoding gene primers. The expected amplicon size (192 bp) was found in all examined samples (Figure-1).

### Oxidant, anti-oxidant, cytokine, acute phase proteins, copper, zinc, iron, TP, albumin, AST, ALT, CPK, BUN, and creatinine concentrations and ratios

**Figure-2:**
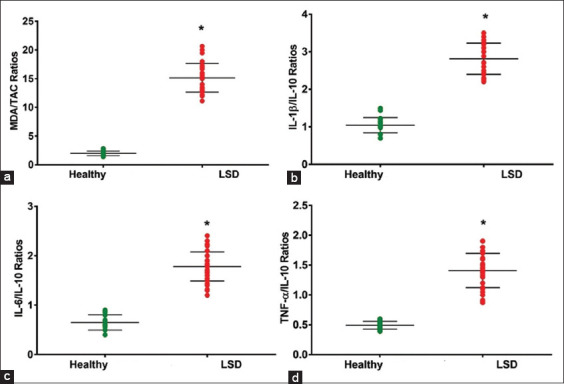
MDA/TAC, IL-1β/IL10, IL-6/IL-10, and TNF-α/IL-10 ratios in cows with LSD. Values expressed with mean ± SD. (a-d) MDA/TAC, IL-1β/IL10, IL-6/IL-10, and TNF-α/IL-10 ratios were significantly elevated in cows with LSD than in healthy cattle. *Indicates significantly different than healthy cattle at p < 0.05. MDA=Malondialdehyde, TAC=Total anti-oxidant capacity, IL=Interleukin, TNF-α=Tumor necrosis factor-alpha, LSD=Lumpy skin disease.

Cattle with LSD had significantly higher serum MDA, IL-1β, IL-6, TNF-α, Hp, and SAA and significantly lower TAC and IL-10 concentrations compared to healthy cattle (p < 0.05; Table-2). Cattle with LSD also had significantly higher serum AST, ALT, CPK, BUN, and creatinine and lower serum copper, zinc, iron, TP, albumin, and globulin concentrations than healthy cattle (p < 0.05; Table-3). Cattle with LSD had higher MDA/TAC ratios (15.15 ± 2.5) than healthy ones (1.9 ± 0.3), higher IL-1β/IL-10 ratios (3.0 ± 0.4) compared to healthy cattle (1.0 ± 0.2), higher IL-6/IL-10 ratios (1.8 ± 0.3) than healthy ones (0.6 ± 0.2) (Figure-2c; p < 0.05), and higher TNF-α/IL-10 ratios in cattle with LSD (1.5 ± 0.2) than healthy cattle (0.4 ± 0.1) (Figure-2a-d; p < 0.05).

**Table-2:** Oxidant/anti-oxidant status, acute phase proteins, and cytokines in healthy cattle and cattle with LSD (mean ± SD).

Variables	Healthy cattle (n = 25)	Cattle with LSD (n = 35)
TAC (mmol/L)	1.5 ± 0.42	0.48 ± 0.11*
MDA (nmol/mL)	3.2 ± 0.36	7.1 ± 0.69*
Hp (mg/mL)	79.9 ± 3.8	99.7 ± 8.2*
SAA (mg/mL)	0.85 ± 0.12	1.27 ± 0.28*
IL-1β (pg/mL)	58.1 ± 6.5	87.8 ± 9.8*
IL-6 (ng/L)	34.8 ± 4.3	55.6 ± 10.1*
TNF-α (ng/L)	25.18 ± 3.2	41.15 ± 7.8*
IL-10 (ng/L)	49.8 ± 5.8	31.8 ± 3.6*

LSD=Lumpy skin disease, SD=Standard deviation, IL=Interleukin, Hp=Haptoglobin, MDA=Malondialdehyde, SAA=Serum Amyloid A, TAC=Total Anti-oxidant Capacity, TNF=Tumor Necrosis Factor,

* p < 0.05

**Table-3:** Serum TP, albumin, globulin, ALT, AST, CPK, urea, creatinine, copper, zinc, and iron concentrations and A/G ratios in healthy cattle and cattle with LSD (mean ± SD).

Variables	Healthy cattle (n = 25)	Cattle with LSD (n = 35)
TP (g/dL)	6.7 ± 0.28	5.5 ± 0.35*
Albumin (g/dL)	3.6 ± 0.19	3.08 ± 0.16*
Globulin (g/dL)	3.1 ± 0.3	2.4 ± 0.36*
A/G ratio	1.16 ± 0.16	1.27 ± 0.2
ALT (U/L)	20.2 ± 2.03	29.5 ± 2.06*
AST (U/L)	62.9 ± 4.6	90.2 ± 5.2*
CPK (U/L)	224.3 ± 6.5	277.4 ± 13.7*
BUN (mmol/L)	1.7 ± 0.46	3.1 ± 0.4*
Creatinine (mg/dL)	1.07 ± 0.15	2.05 ± 0.28*
Copper (mmol/L)	16.9 ± 1.06	11.9 ± 1.08*
Zinc (mmol/L)	24.08 ± 1.4	15.4 ± 1.04*
Iron (mg/dL)	92.3 ± 3.7	57.9 ± 7.2*

LSD=Lumpy skin disease, SD=Standard deviation, A/G=Albumin/Globulin, ALT=Alanine Aminotransferase, AST=Aspartate Aminotransferase, CPK=Creatine Phosphokinase, TP=Total Protein,

* p < 0.05

### Correlations between oxidants, anti-oxidants, trace elements, inflammatory, and anti-inflammatory cytokines

In cattle with LSD, serum MDA concentrations were inversely correlated with TAC (r = −0.42; p < 0.05), zinc (r = −0.52; p < 0.05), and copper (r = −0.45; p < 0.05) concentrations, but were not correlated with iron concentrations. Serum IL-10 concentrations were negatively correlated with IL-1β and IL-6 concentrations (r = −0.40; p < 0.05; r = −0.51; p < 0.05), respectively, but were not associated with TNF-α concentrations. Serum Hp concentrations were positively correlated with IL-1β and TNF-α concentrations (r = 0.40; p < 0.05; r = 0.46; p < 0.05), respectively. Serum SAA concentrations were positively associated with TNF-α (r = 0.44; p < 0.05).

In healthy cattle, serum MDA concentrations were not correlated with TAC, zinc, copper, and iron concentrations. Serum IL-10 concentrations were correlated with IL-1β, IL-6, and TNF-α concentrations. Serum Hp and SAA concentrations were not correlated with IL-1β, IL-6, and TNF-α concentrations.

### Univariate analysis for pro-oxidant and anti-oxidant status, zinc, copper, and iron concentrations in cattle with LSD

Affected cattle with zinc concentrations <22.2 µmol/L were more likely to have high MDA and low TAC concentrations (OR = 3.2; 95% CI = 1.5–11.1; OR= 2.4; 95% CI = 1.1–6.4), respectively (Table-4; p < 0.05). The likelihood of elevated IL-1β and IL-6 and decreased IL-10 concentrations was associated with low zinc concentrations (OR = 4.5; 95% CI = 1.8–18.6; OR = 2.6; 95% CI = 1.1–8.9; OR = 5.2; 95% CI = 2.4–20.2; p < 0.05), respectively, but TNF-α were not (Table-4). Cattle with copper concentrations <15.2 µmol/L were more likely to have increased MDA concentrations (OR = 5.2; 95% CI = 2.4–16.8; p < 0.05) but were not associated with the likelihood of low TAC concentrations (Table-4). The likelihood of elevated IL-1β and decreased IL-10 concentrations was associated with low zinc concentrations in cattle with LSD (OR = 4; 95% CI = 1.5–19.2; OR = 3.6; 95% CI = 1.1–12.3; p < 0.05), but IL-6 and TNF-α were not (Table-4). Affected cattle with iron concentrations <85.3 mg/dL were more likely to have elevated TNF-α concentrations (OR = 3.4; 95% CI = 1.01–12.6; p < 0.05); however, the likelihoods of increased IL-1β and IL-6 and decreased IL-10 concentrations were not associated with iron concentrations <85.3 mg/dL in the affected cattle with LSD (Table-4).

**Table-4:** Univariate analysis of low trace element concentrations in cattle with LSD.

Variables		Range	OR	95% CI	p-value
MDA (nmol/mL)	Zinc (µmol/L)	22.2–26.2	Reference		0.03
		<22.2	3.2*	1.5–11.1	
TAC (mmol/L)			2.4*	1.1–6.4	0.04
IL-1β (pg/mL)			4.5*	1.8–18.6	0.03
IL-6 (ng/L)			2.6*	1.1–8.9	0.04
TNF-α (ng/L)			0.8	0.4–3.2	0.5
IL-10 (ng/L)			5.2*	2.4–20.2	0.01
MDA (nmol/mL)	Copper (µmol/L)	15.2–18.4	Reference		0.02
		<15.2	5.2*	2.4–16.8	
TAC (mmol/L)			1.04	0.8–3.6	0.4
IL-1β (pg/mL)			4*	1.5–19.2	0.02
IL-6 (ng/L)			0.5	0.7–7.5	0.2
TNF-α (ng/L)			0.65	0.8–6.8	0.4
IL-10 (ng/L)			3.6*	1.1–12.3	0.03
MDA (nmol/L)	Iron (mg/dL)	85.3–97.2	Reference		
		<85.3	1.4	0.6–6.8	0.3
TAC (nmol/L)			0.7	0.9–8.9	0.4
IL-1β (pg/mL)			1.1	0.8–4.8	0.3
IL-6 (ng/L)			0.82	0.5–5.6	0.4
TNF-α (ng/L)			3.4*	1.01–12.6	0.03
IL-10 (ng/L)			0.66	0.4–3.8	0.5

LSD=Lumpy skin disease, CI=Confidence interval, IL=Interleukin, MDA=Malondialdehyde, OR=Odds ratios, TAC=Total anti-oxidant capacity, TNF=Tumor necrosis factor,

* p < 0.05

### Risk analysis of LSD oxidative stress on sex, breed, and age

Cows with LSD were more likely to have serum MDA concentrations >3.8 nmol/L and TAC concentrations <0.9 mmol/L than bulls (OR = 5.8; 95% CI = 2.4–18.5 OR = 8.6; 95% CI = 1.2–30.4), respectively (Table-5; p < 0.05). Exotic mixed breed cattle with LSD were more likely to have serum MDA concentrations >3.9 nmol/L than local breed ones (OR = 12.4; 95% CI = 1.8–65.6); however, TAC concentrations were not different between breeds (Table-5; p > 0.05) no significance between age (Table-5; p > 0.05).

**Table-5:** Univariate risk analysis of LSD oxidative stress on sex, breed and age.

MDA>3.8 nmol/mL			

Variables	OR	95% CI	p-value
Sex			
Bulls	Reference		0.04
Cows	5.8*	2.4−18.5	
Breeds			
Local	Reference		0.02
Exotic mixed	12.4*	1.8−65.6	
Age			
3 years	Reference		
2 years	1.1	0.3−8.7	0.4

**TAC<0.9 mmol/L**			

**Variables**	**OR**	**95% CI**	**p-value**

Sex			
Bulls	Reference		0.01
Cows	8.6*	1.2−30.4	
Breeds			
Local	Reference	0.3−0.8	0.2
Exotic mixed	0.9	0.4−3.8	
Age			
3 years	Reference		
2 years	1.8	0.7−11.6	0.3

CI=Confidence Interval, MDA=Malondialdehyde, OR=Odds Ratio, TAC=Total anti-oxidant capacity,

* p < 0.05

### Sensitivity and specificity of Hp and SAA concentrations in cattle with LSD

**Figure-3:**
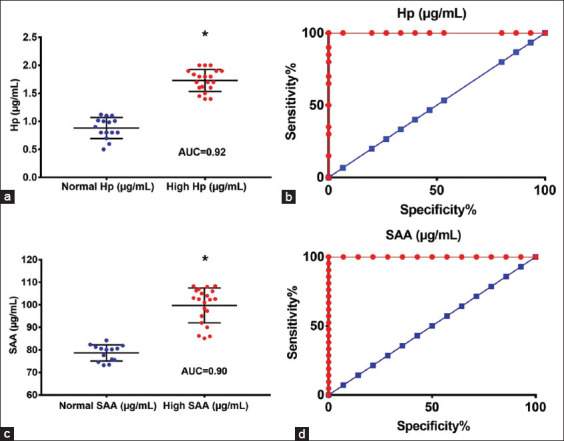
Receiver operating characteristic data for Hp and SAA in cattle with LSD based on 5–95% CI. (a and b) Serum Hp concentrations >1.4 μg/mL had 95% sensitivity and 100% specificity to predict LSD in cattle (AUC = 0.92; p < 0.01) (c and d) SAA concentrations >85.5 μg/mL had 90% sensitivity and 100% specificity to predict LSD in cattle (AUC = 0.90; p < 0.01). Hp=Haptoglobin, SAA=Serum amyloid A, LSD=Lumpy skin disease, CI=Confidence interval, AUC=Area under the curve.

Serum Hp concentrations >1.4 μg/mL had 95% sensitivity and 100% specificity (area under the curve [AUC] = 0.92; p < 0.01; Figures-3a and b) and SAA concentrations >85.5 μg/mL had 90% sensitivity and 100% specificity (AUC = 0.90; p < 0.01; Figures-3c and d) to predict LSD in cattle.

### Gross and histopathological examination in cattle with LSD

**Figure-4:**
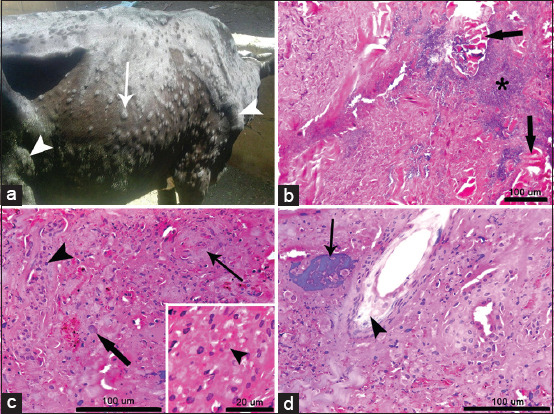
Gross and histologic images of skin biopsy from a cow with LSD. (a) Cow with LSD showing skin nodules of up to 5 cm in diameter (white arrow) as well as enlarged pre-scapular and pre-femoral lymph nodes (white arrowheads). (b) Cutaneous nodule showing extensive necrosis in epidermal and dermal layers with Zenker’s necrosis in the dermal muscles (2 arrows) and mononuclear cells aggregation (asterisk). (c) Central part of a cutaneous nodule showing extensive necrosis in epidermal and dermal layers, mononuclear cell aggregation (arrowhead), macrophage activation (thick arrow), and severe vasculitis (thin arrow). Inset: Eosinophilic intracytoplasmic inclusion body in a keratinocyte (arrowhead). (d) Central part of a cutaneous nodule showing a giant cell with phagocytosed tissue debris (arrow) and perifollicular edema (arrowhead). LSD=Lumpy skin disease.

Dermal nodules in the affected cattle were 2–5 cm in size, well-circumscribed, round, slightly raised, and firm (Figure-4a). Microscopically, the central part of the nodules showed extensive necrosis of epidermal and dermal layers, with heavy infiltration with mononuclear inflammatory cells, including macrophages and lymphocytes. In addition, extensive and severe vasculitis was evident. Eosinophilic intracytoplasmic inclusion bodies were observed in keratinocytes of all affected cows (Figures-4b and c). There were perifollicular edema and giant cells which phagocytosed tissue debris (Figure-4d).

## Discussion

In this study, we investigated oxidative, anti-oxidant, and inflammatory indicators as well as selected biochemical markers in cattle with LSD. We showed that cattle with LSD were in a pro-oxidant state characterized by increased MDA and reduced TAC and microelements concentrations. There was also evidence of systemic pro-inflammatory response, with increased concentrations of pro-inflammatory and decreased concentrations of anti-inflammatory cytokines. Elevations of acute phase proteins provided further confirmation of the systemic impact of LSD in these animals. These findings indicate that systemic oxidative injury and inflammation are central to the pathogenesis of LSD. Our results are aligned with previous studies on the oxidative and inflammatory nature of LSD in cattle [8, 9]. However, this is the first study to evaluate microelements known to have anti-oxidant and anti-inflammatory properties in cattle with LSD.

It is likely that increased serum MDA and decreased TAC concentrations in the affected cattle of this study were a direct consequence of LSDV triggering oxidative cascades and downregulating anti-oxidant mechanisms (e.g., low copper, zinc, and TAC concentrations), ultimately leading to tissue injury [23].

High concentrations of IL-1β, IL-6, and TNF-α in the affected cows of this study confirms the systemic inflammatory nature of LSD, likely in part triggered by dermal macrophages and lymphocytes [9, 10], while decreased IL-10 concentrations worsen clinical disease [24, 25].

Zinc, copper, and iron are co-factors for anti-oxidant enzymes (e.g., superoxide dismutase and catalase) and their decrease impairs anti-oxidant capacity, which indirectly promotes cell membrane oxidative injury [26]. This was evident with low zinc concentrations that were associated with reduced anti-oxidant capacity in cattle affected with LSD. Copper concentrations were not associated with anti-oxidant capacity but rather with pro-oxidant markers. Our findings were in line with a previous study where cattle with LSD had low zinc, copper and iron concentrations [27]. It is possible that tissue sequestration of copper, iron, and zinc by anti-oxidant enzymes contributed to their lower concentrations in the sick cattle of this study [7, 28, 29].

An increased acute phase protein (Hp and SAA) in cattle with LSD was not surprising and consistent with prior studies confirming the hepatic response to systemic inflammation, likely mediated by cytokines [30]. Hp and SAA concentrations were also good biomarkers of cattle in cows [12, 31].

Cattle with LSD had elevated AST, ALT, CPK, urea, and creatinine concentrations, suggesting that this virus, directly or indirectly, causes liver, muscular, and kidney injury [32], which may be relevant to disease progression and weight loss. Decreased TP, albumin, and globulin concentrations further confirm hepatic involvement but could also overlap with the negative acute phase protein response.

In the current study, cows and exotic mixed breeds were at a higher risk of LSD oxidative stress compared to bulls and local breeds. However, age was not significant among cattle with LSD in this study. It is plausible that genetic variations of innate immunity are central to LSD pathogenesis based on sex and breed [33, 34].

Epidermal and dermal necrosis, dermal necrotizing vasculitis, infarction, edema, diffuse infiltration with inflammatory cells, and intracytoplasmic inclusion bodies were consistent findings in the affected cattle with LSD of this study, supporting the diagnosis of this condition [13, 14, 35].

## Conclusion

LSD is a highly infectious endemic viral disease of cattle in Africa, the Middle East, and Turkey and spreading to other countries. Systemic pro-oxidant and pro-inflammatory states from this study support the role of these processes in the pathogenesis of LSD. Reduced copper, zinc, and iron concentrations further confirm the anti-oxidant value of these microelements in cattle with LSD. Elevated SAA and Hp concentrations could predict cattle with LSD. This study provided additional insight into the pathogenesis of LSD and our results could have clinical implications, in particular in the implementation of anti-oxidant therapies to reduce disease severity.

## Authors’ Contributions

HH, AK, and AA: Designed the study. AK, MN, and AA: Performed the research and drafted the manuscript. AnA: Performed the gross and histopathological examination. HH and RT: Followed-up the experiments and edited the manuscript. RT: Revised the manuscript for publication. All authors have read and approved the final manuscript.
